# Editorial: Methanotrophs: Diversity, Environmental Relevance and Applications

**DOI:** 10.3389/fmicb.2021.796861

**Published:** 2021-11-29

**Authors:** Francisco J. Cervantes, Sarahi L. Garcia, Sari Peura, Nagamani Balagurusamy

**Affiliations:** ^1^Laboratory for Research on Advanced Processes for Water Treatment, Engineering Institute, Campus Juriquilla, Universidad Nacional Autónoma de México (UNAM), Querétaro, Mexico; ^2^Department of Ecology, Environment and Plant Sciences, Science for Life Laboratory, Stockholm University, Stockholm, Sweden; ^3^Department of Forest Mycology and Plant Pathology, Science for Life Laboratory, Swedish University of Agricultural Sciences, Uppsala, Sweden; ^4^Laboratorio de Biorremediación, Facultad de Ciencias Biológicas, Ciudad Universitaria de la Universidad Autónoma de Coahuila, Torreón, Mexico

**Keywords:** methanotrophs, distribution, metabolic diversity, genomics, cultivation method

Greenhouse gas (GHG) emissions are a serious environmental menace, which challenge our existing living conditions. Methane is the second largest GHG, and is derived from both anthropogenic activities and natural ecosystems. Methanotrophs, microorganisms with the capacity to oxidize methane under oxic or anoxic conditions, are recognized as key actors regulating the methane emissions in terrestrial and aquatic environments.

This Research Topic served as a platform to disseminate recent scientific knowledge on methanotrophs. Moreover, it highlights the importance of methanotrophy, both in natural ecosystems as well as in potential biotechnological applications for mitigation. The research in this topic, makes it clear that there are many variables controlling methanotrophy. Factors such as availability of electron- donors, -acceptors, conditions like pH, temperature, etc. along with types of microbial diversity and their interactions play a major role in defining the strength of various sources and sinks for methane (Figure 1).

**Figure 1:**
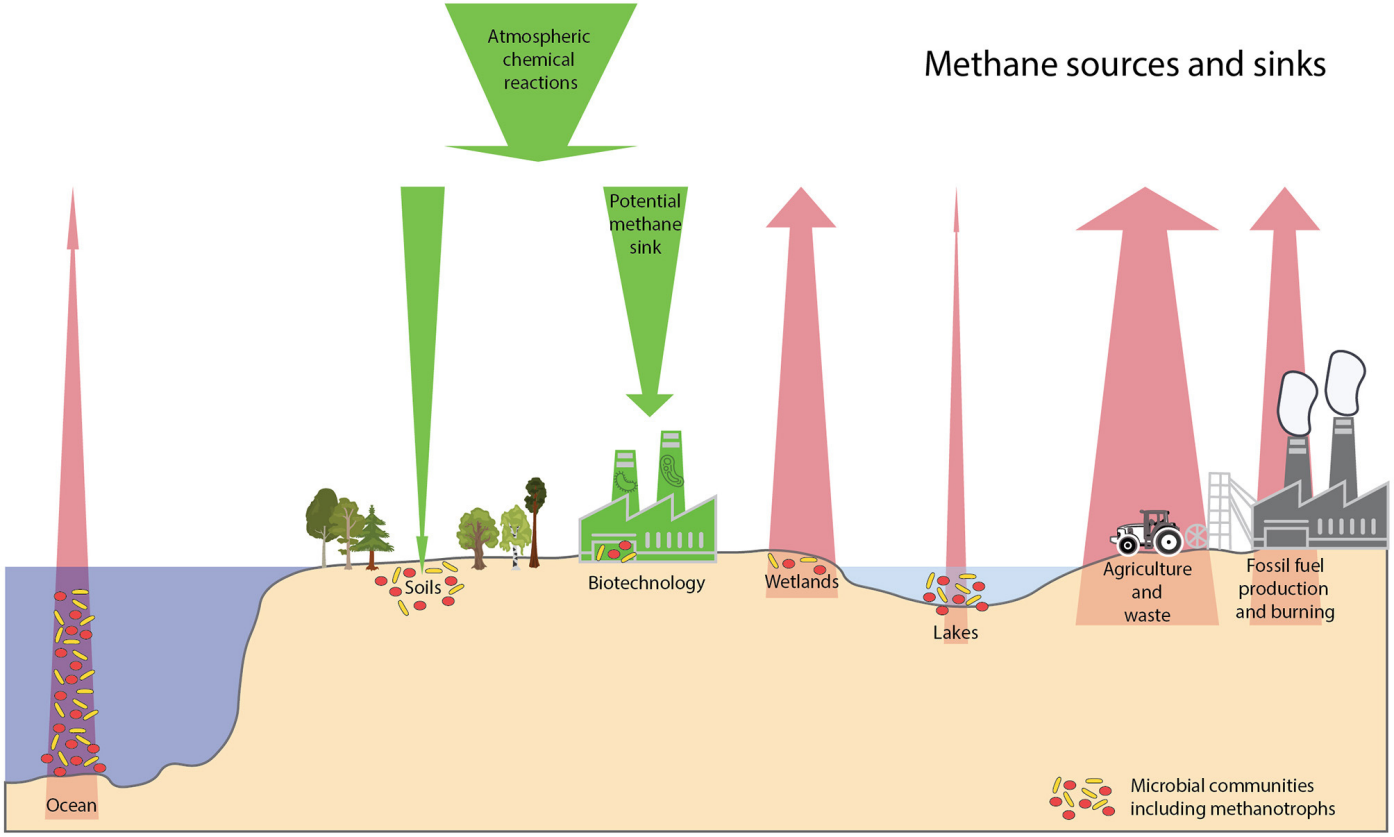
Schematic diagram on the different sources and sinks of methane. Sizes of trees, industries and microbes are not to scale. Red arrows show the sources of methane, agriculture and waste being the biggest anthropogenic source with about 217 teragrams of CH4 per year (https://doi.org/10.5194/essd-12-1561-2020). Wetlands are the biggest natural sources of methane emissions with about 181 teragrams of CH4 per year. Other minor natural emissions of methane are inland waters, oceans, termites, wild animals, permafrost and vegetation. Methanotrophs and their metabolic partners can act as a methane biofilter in some environments such lakes and ocean depending on the microbial composition and the availability of electron acceptors. Green arrows show the sinks of methane, atmospheric chemical degradation being the biggest sink with 518 teragrams of CH4 per year. Diverse methanotrophs could be cultivated and studied and further used in biotechnological applications to produce high-value natural products of interest to humans (https://doi.org/10.3389/fmicb.2021.678057).

Picone et al. reported characterization of a novel thermophilic methanotroph belonging to the genus *Methylacidimicrobium* (*Methylacidimicrobium thermophilum* AP8), which was isolated from a geothermal area located in Pantelleria Island (Italy). The ability of *M. thermophilum* AP8 to oxidize methane and hydrogen in volcanic environments, where oscillation in oxygen concentrations and the availability of substrates (e.g., CH_4_ and H_2_) might occur, suggests that this new methanotroph may play an important role in regulating methane emissions from geothermal zones.

In another interesting paper, Picone et al. discovered the presence of two verrucomicrobial Metagenome Assembled Genomes (MAGs) based on analysis of DNA extracted from the soil of the same geothermal area in Pantelleria Island, Italy. After extensive analysis of one of the MAGs, they proposed the name “*Candidatus* Methylacidithermus pantelleriae” gen. nov. sp. nov. for it. Genes encoding for particulate methane monooxygenase (pMMO) and a XoxF-type methanol dehydrogenase (MDH) were identified. Further analyses revealed the biochemical pathways used by this methanotroph. Additionally, the authors concluded that “*Ca*. Methylacidithermus pantelleriae” might be capable of nitric oxide reduction, but genes for dissimilatory nitrate reduction and nitrogen fixation were not identified. The discovery of this new MAG expands the diversity and metabolism of verrucomicrobial methanotrophs.

Another fascinating paper included in this Research Topic was published by Martin et al., who used shotgun metagenomics data to explore the diversity and distribution of methanotrophs in 40 oxygen-stratified water bodies in boreal and subarctic environments in Europe and North America. They found that gammaproteobacterial methanotrophs (order *Methylococcales*) generally dominated the methanotrophic communities throughout the water columns. *Candidatus* Methylumidiphilus, belonging to *Methylococcales*, was present in all the studied water bodies and dominated the methanotrophic community in lakes with a high relative abundance of methanotrophs. Alphaproteobacterial methanotrophs were the second most abundant group of methanotrophs. The authors also found that relative abundances of anaerobic methanotrophs, *Candidatus* Methanoperedenaceae and *Candidatus* Methylomirabilis, were strongly correlated, suggesting a potential co-metabolism. The experimental evidence also suggested that these anaerobic methanotrophs could be active even in the oxic layers. This study ratifies the importance of O_2_ and CH_4_ in determining the composition of methanotrophic communities and that the variety and distribution of freshwater methanotrophs are controlled by specific conditions prevailing in lakes.

Rahalkar et al. developed a distinctive method for the cultivation of methanotrophs from Indian rice field samples located in the tropical region. This is an important contribution since a total of 29 strains were obtained. These could be used as models for studying methane mitigation from rice fields and for environmental and biotechnological applications. The approach yielded methanotrophs from seven genera belonging to three major groups: Type Ia (*Methylomonas, Methylomicrobium*, and *Methylocucumis*), Type Ib (*Methylocaldum* and *Methylomagnum*), and Type II (*Methylocystis* and *Methylosinus*). The study employed 16S rRNA gene-based next-generation sequencing (NGS) for identification of methanotrophic community structure in three rice rhizosphere samples.

Finally, our Research Topic is complemented by a comprehensive review by Guerrero-Cruz et al. who provide a synopsis of key aspects of ecology, physiology, metabolism, and genomics to understand the impact of methanotrophs in the mitigation of methane emissions to the atmosphere. Moreover, this review presents a particular focus on processes under microaerophilic and anaerobic conditions for both aerobic and anaerobic methane oxidizers. The article also highlights the current and upcoming applications of methanotrophs for mitigating methane releases from wastewater treatment facilities and other biological functions.

The articles significantly contribute to our understanding of the role of methanotrophs in different environments such as lakes, volcanic soils, sediments, and rice fields. We hope that this Research Topic will support policymakers to identify and decide on the methane mitigation strategies to be adopted on a local, national and global scale.

## Author Contributions

FC drafted the manuscript, which all authors contributed to writing and editing. SG illustrated the figure in consultation with SP, which was revised and approved by all authors. NB uploaded the final version and responded to all the comments of the reviewers and submitted it after approval by all co-authors. All authors have made a substantial, direct, and intellectual contribution to this publication.

## Funding

FC was supported by the National Council of Science and Technology of Mexico (CONACYT, Grant Frontiers in Science No. 682328) and by Universidad Nacional Autónoma de México (UNAM, PAPIIT Grant TA100120). SG and SP were supported by Science for Life Laboratory. NB acknowledges the support of the Universidad Autónoma de Coahuila (proyectos semillas DIP-UADEC C01-2021-105).

## Conflict of Interest

The authors declare that the research was conducted in the absence of any commercial or financial relationships that could be construed as a potential conflict of interest.

## Publisher's Note

All claims expressed in this article are solely those of the authors and do not necessarily represent those of their affiliated organizations, or those of the publisher, the editors and the reviewers. Any product that may be evaluated in this article, or claim that may be made by its manufacturer, is not guaranteed or endorsed by the publisher.

